# Gender underlies the formation of STEM research groups

**DOI:** 10.1002/ece3.6188

**Published:** 2020-04-01

**Authors:** Denon Start, Shannon McCauley

**Affiliations:** ^1^ Center for Population Biology UC Davis Davis CA USA; ^2^ Department of Biology University of Toronto Mississauga Mississauga ON Canada

**Keywords:** academia, diversity, science workforce, STEM

## Abstract

Research groups are the cornerstone of scientific research, yet little is known about how these groups are formed and how their organization is influenced by the gender of the research group leader. This represents an important gap in our understanding of the processes shaping gender structure within universities and the academic fields they represent. Here, we report the results of an email survey sent to department chairs and discipline‐specific listservs. We received responses from 275 female and 175 male research group leaders. Most respondents were biologists (*n* = 328) but psychology (*n* = 27), chemistry (*n* = 16), physics (*n* = 32), and mathematics (*n* = 30) were also relatively well represented. We found that men were self‐reported as overrepresented in research groups in the physical sciences, particularly at later career stages. Within biology, male and female group leaders reported supervising a disproportionate number of same‐gender trainees (students and postdoctoral fellows), particularly early in their careers. These self‐reported patterns were driven primarily by gender‐based differences in the pool of students applying to their research groups, while gender differences in acceptance rates played a seemingly smaller role. We discuss the implications of our results for women continuing into the professoriate and for the recruitment of young scientists into research groups.

## Graphical abstract

Gender differences are pervasive in STEM, yet we know relatively little about the factors structuring gender differences in research groups—the fundamental unit of scientific investigation. We show that gender of the applicant pool and the principal investigator interact to shape gender representation in research groups. Our study has important implications for the continuation of women in STEM fields and for the performance of science as a whole.

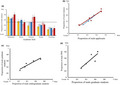

## 1 INTRODUCTION

The advancement of basic and applied sciences, largely through academic pursuits, is central to our understanding of the world and the advancement of our societies. The cornerstone of scientific research is the research group, a principal investigator (PI) along with their students (graduate and undergraduate) and postdoctoral fellows (PDs). These research groups train new scientists who enter the scientific workforce in a variety of capacities both inside and outside of academia, and research conducted in these groups provides valuable scientific contributions (Pezzoni, Mairesse, Stephan, & Lane, 2016). Despite the central role that academic laboratories play in scientific training for STEM fields, the patterns of gender composition in research (laboratory) groups have rarely been investigated (but see: Sheltzer & Smith, 2014).

Differences in gender composition in a given STEM field or research group can come about through two distinct, though not mutually exclusive, mechanisms. A gender may be underrepresented because they apply in fewer numbers (1; application differences) or because fewer applicants of that gender are chosen to join the research group (2; acceptance differences). These two mechanisms are not necessarily independent (e.g., women apply less and are chosen less as PDs in math‐intensive fields) and can have complex underlying causes (Ceci & Williams, 2010, 2011). We suggest that understanding the relative importance of these two mechanisms and their underlying cause(s) can help fields and research groups understand and reflect on differences in gender composition in STEM fields.

Patterns of application and acceptance differences between genders have been attributed to several underlying causes. Early career aspirations, perceptions of the need for innate ability and stereotype threat (Leslie, Cimpian, Meyer, & Freeland, 2015), choice of undergraduate major, and personal aspirations (e.g., work‐life balance) have all been proposed as explanations for the lack of female applicants in particular fields (e.g., math and physics; Ceci & Williams, 2011; Morgan, Gelbgiser, & Weeden, 2013; Sadler, Sonnert, Hazari, & Tai, 2012). Implicit and explicit bias of professors has similarly been suggested to affect gender differences in acceptance rates from the applicant pool (Ceci & Williams, 2011; Moss‐Racusin, Dovidio, Brescoll, Graham, & Handelsman, 2012), with the former likely being more prevalent (Ceci & Williams, 2010). Each of these causes can independently or jointly underlie the ultimate patterns of gender differences among fields, but do not provide clear insights into the finer scale structure of research groups. This is vital to understand because gender ratios at the research group level can have a strong effect on the gender composition of the researchers in the field whenever male and female PIs are not equally represented. The structure of these units influences the gender composition at each step of the training process up to the point at which individuals seek independent positions as faculty or in other research capacities. Therefore, the pipeline that ultimately leads to independent scientists necessarily passes through these research groups. Past work has shown that while PI gender and status are related to research group gender composition, whether these factors manifest themselves by influencing decisions to apply or the acceptance rates of applicants is unknown (Sheltzer & Smith, 2014). While our study cannot distinguish the underlying reasons for application or acceptance differences, it can shed light on which of these processes acts most strongly to structure laboratory groups.

Outside of the highest‐profile research groups (Sheltzer & Smith, 2014), whether group leader gender is associated with gender ratios within their groups is poorly known. Previous research suggests that the gender of the PI and their level of achievement or experience may explain some of the variation in gender among research groups (Sheltzer & Smith, 2014). Importantly, the vast majority of students are trained in laboratories that are run, not by these highest status PIs (as defined by Sheltzer & Smith, 2014), but by PIs who have not achieved the same status. Therefore, to explain gender dynamics within fields more broadly, we require an understanding of laboratory formation in typical rather than high‐status laboratories. Our lack of understanding on the underlying cause of this pattern precludes the ability to thoroughly understand patterns underlying gender differences among the majority of research groups training future scientists.

We aimed to investigate patterns of gender structure in scientific research groups by simultaneously considering applicants to these research groups and current trainees across academic stages (we consider undergraduate students, graduate students, and postdocs (hereafter: PDs) to be trainees but at different academic stages). We used survey techniques to consider patterns at two scales of organization: across scientific fields and within biology (the field for which we had the greatest representation). Specifically, we sent online surveys (Appendix S1, Table S1) to department chairs (science‐based departments in Canada) and asked them to forward the link to all professors in their department, then also disseminated the same survey through various subject‐specific email lists (largely biology related) which reach an international pool of scientists. We discuss potential limitations and biases inherent to this approach below. Self‐reported patterns suggest that there were fewer women in research groups in the physical sciences (math, physics, chemistry), and that these differences were largely driven by differences in the gender composition of the applicant pool. Conversely, within biology, variation in the self‐reported gender composition of research groups was jointly determined by application and acceptance differences. Reported patterns of applicant gender were largely determined by PI gender and experience. In sum, application and acceptance differences both contribute to the over‐/underrepresentation of women in particular fields and research groups, although application differences seem to dominate.

## 2 METHODS

### 2.1 Data collection

We collected data on the gender composition of academic laboratory groups (PI, PDs, graduate and undergraduate students), and potential predictors we hypothesized might affect the gender composition of laboratory groups using a short online survey. Note that while laboratory technicians and staff scientists can be invaluable in many research groups, we did not include these personnel in our survey as we were focused on researchers in the classic academic progression from undergraduate to graduate student to PD to PI. We asked twenty‐one questions with either short (e.g., institutional/departmental affiliation) or numerical (e.g., gender ratio of male and female graduate students within a laboratory group) answers then allowed participants to provide any other comments that they thought we may find useful (Appendix S1, Table S1). Note that when inquiring about gender ratios of applicants, we asked that respondents only include legitimate applications, rather than generic form‐style email applications that are frequently mass distributed. By comparing the rates at which men and women apply to a given research group with current gender representation in that group, we can better understand whether patterns of laboratory gender composition are driven by gender‐biased applications or acceptance rates. To maximize responses and promote honest answers, we chose to make our survey anonymous, precluding an analysis of the achievements of the PI as a factor in determining gender differences (e.g., Pezzoni et al., 2016). Survey methods are likely to result in a biased subset of answers, for example those people choosing not to respond may include the professors with the greatest (implicit or explicit) gender bias. We discuss the issue of selection bias in our survey in greater detail below.

Our goal was the widest possible dissemination of our survey with a focus on biology in Canada, our field and region of study. We circulated the survey in two ways: (a) by emailing Canadian department heads (chairs) of major departmental divisions (mathematics, physics, chemistry, biology, psychology) and asking them to forward our survey to other faculty in their department and (b) by sending mass emails through subject listservs and email lists. Note that we received >70% of all responses from the United States and >95% of responses from North America, but from a wide variety of universities. One potential reason is that the survey was only sent in English, making it potentially less accessible to faculty at francophone universities in Canada. These universities and even departments within universities are likely to differ in many ways, including in size, research focus, and recruiting policies.

Participants received a link to our SurveyMonkey (SurveyMonkey© 2017) questionnaire (Appendix S1, Table S1), a statement that we were generally investigating patterns of gender in academia (Appendix S1), and the required human ethics documentation. Following the dissemination of our survey, we allowed participants more than 4 weeks to respond, ending data collection when we received no responses for three consecutive days.

Note that while surveys can produce biased subsets of respondents, they are used in similar studies (e.g., Riffle et al., 2013). Biases are likely to weaken or strengthen any observed patterns (e.g., men have some particular percentage of graduate students), but not affect qualitative differences (e.g., men have more male graduate students; Ceci & Williams, 2010; Ceci & Williams, 2011; Sheltzer & Smith, 2014). Additionally, given the diversity of ways in which applications are handled across institutions and even across departments within institutions, surveying individuals PIs is the most feasible way to generate adequate and comparable data across a large number of STEM fields and institutions. On the other hand, clear opportunities for bias arise in our survey methods. While we recognize that this survey relies on faculty memories, which may be imperfect, by restricting these recollections to candidates who would be seriously considered we assume that faculty estimates of applicant numbers are close to accurate and unbiased with respect to the questions we address here (there is a further discussion of recall bias below). We are also assuming that faculty are correctly assessing the gender of applicants, which may not be the case, particularly when the applicant is from another culture or has a name from a language unfamiliar to the PI. Nonetheless, we discuss the potential for biased subsets of respondents (e.g., fewer responses from more biased faculty), recall bias (e.g., more recollection of male or female applicants), and social desirability bias (e.g., under‐ or overreporting the male application rates) in the discussion.

### 2.2 Data proofing

Before beginning our analyses, we extensively proofed the data. First, we only retained data for individuals that completed the survey (>90% of all individuals). Note that we do not know the proportion of respondents from listservs versus emails to department chairs, but only Canadian department chairs were emailed while only ~25% of respondents were at Canadian institutions. This then suggests that the vast majority of responses were from individuals contacted through listservs, and the survey is likely more representative of American than Canadian institutions. Next, we removed all answers that were clearly fraudulent (e.g., Trump University, *n* = 2). We then removed all instances of non‐numerical responses to quantitative questions (e.g., how many male graduate students), replacing these values with NAs. Finally, we removed PIs who self‐identified as gender nonbinary (*n* = 1). While we recognize the potentially unique position of nonbinary PIs, the low number of nonbinary participants precluded a thorough statistical analysis. However, we will note here that the research group of the nonbinary PI contained an equal number of male and female graduate students. We also note that we inquired as to the gender, rather than the biological sex, of respondents and trainees. While not a goal of our study, we certainly acknowledge the potential important of sex versus gender differences in academia.

We have included summary statistics of survey results in Appendix S2. We next assigned each PI to one of five fields based on their departmental affiliation (psychology, biology, chemistry, physics, mathematics; Appendix S2, Table S3). In subsequent analyses, we removed respondents from fields that were not biology, psychology, mathematics, physics, or chemistry, because other fields were very sparsely represented (Table S3; total *n* = 17 yielding a true sample size of *n* = 433).

We calculated percentage of males for each academic category (e.g., percentage of male applicants to a research group). In all cases, gender ratios are represented as the proportion of males (males/total group) within a laboratory group. Because proportions do not account for sample size (e.g., from the perspective of statistical error 1:1 is not the same as 10:10), we repeated all below regression analyses while weighting each point (proportion) by its sample size, hence accounting for differences in the amount of data composing each proportion. We analyzed this data set in two ways, investigating patterns of gender both among fields and then within biology groups.

### 2.3 Among‐field analyses

We began by testing for patterns of gender composition of trainees within laboratory groups and applicants across academic fields. We first used academic field to estimate the proportion of male graduate applicants in a general linear regression (GLM) with a logit link function, including academic experience of PIs and PI gender as random effects. We repeated this analysis for undergraduate trainees (research students), graduate trainees, PD applicants, and PD trainees. Note that we did not include institution or department as a random effect because most of our responses were from different institutions, and because institution did not predict any measure of gender representation at any stage (using GLM all *p* > .25).

We next aimed to link the gender of applicants and trainees across academic stages and fields. We used rank‐order correlations to predict the percentage of male trainees using the percentage of male applicants separately for graduate students and PDs. We then tested for the scaling of gender representation (percentage) across academic stages, using the above model to estimate the percentage of male graduate trainees from the percentage of male undergraduate research trainees and to estimate the percentage of male PDs from the percentage of male graduate trainees. In order to test for consistent gender patterns across academic stages, we regressed academic stage against the percentage of males using a logistic regression, while including field of study as a random effect.

### 2.4 Within‐field analyses for biology

We tested for gender patterns within fields by analyzing data from biology laboratory groups, the field for which we had the most data. We first tested for differences in the percentage of male trainees and applicants between male and female PIs using generalized linear regressions with logit link functions, repeating this analysis separately for each academic stage. We next aimed to determine whether any differences in the gender ratios of trainees and applicants were a direct result of PI gender or rather owing to differences in the gender composition of applicants. We accomplished this by using a linear regression with the percentage of male graduate students as the response variable and the percentage of male applicants and PI gender as predictors. We then used another linear regression to test for factors potentially controlling the gender composition of applicants, regressing academic experience of applicants and PI gender against the percentage of male applicants in a fully interactive model. In all above analyses, significance was calculated using log‐likelihood ratios, with likelihoods determined from the maximum likelihood solution. All analyses were conducted in R (R core team), using the “*lme4*” (Bates, Mächler, Bolker, & Walker, 2014), and “*car*” (Fox & Weisberg, 2011) packages.

We then attempted to infer causal links and join our analyses together using structural equation modeling (SEM). We began by assuming that PI gender, academic experience of PIs, and the gender makeup of applicants affected the gender composition of trainees but not vice versa (but see discussion for arguments against this assumption). We therefore fit all SEMs using regression rather than covariance, although relaxing these assumptions yielded qualitatively identical results. Before constructing SEMs, we standardized all independent and dependent variables by subtracting the mean and dividing by the standard deviation. We began our analyses with the simplest possible model by linking PI gender, the percentage of male applicants, and academic experience to the percentage of male trainees. We then sequentially added links, selecting the best‐fit model using AIC and calculating the significance for each link using log‐likelihood ratio estimates of standard error. All SEM analyses were performed in R (R core team) using the “*lavaan*” (Rosseel, 2012) package.

## 3 RESULTS

### 3.1 Gender patterns among fields

**Figure 1:**
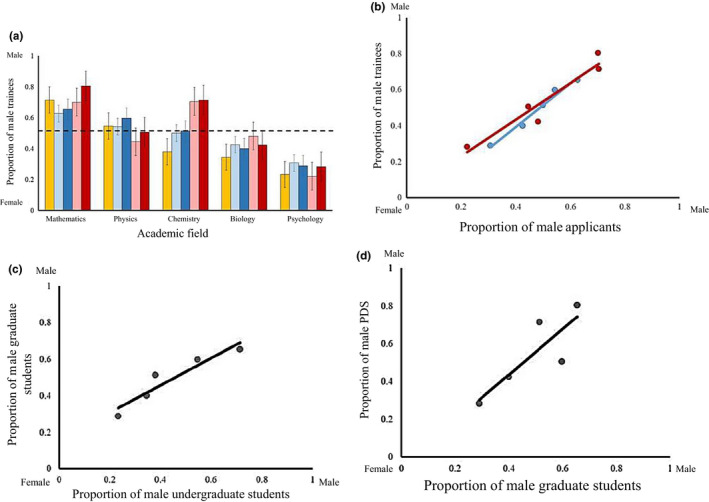
Patterns of gender of survey respondents among academic fields. (a) The proportion of male trainees across academic fields and stages. Yellow bars represent undergraduate trainees. Blue and red bars represent graduate students and PDs, with light and dark bars showing applicants and trainees, respectively. We have arranged the fields from those perceived to be most to least math intensive (or from “hard” to “soft” sciences). The black dashed line represents equal number of male and female trainees/applicants. Error bars represent one *SE* from a logistic regression. (b) The proportion of male applicants is related to the proportion of male trainees for both graduate students (blue) and PDs (red). (c) The proportion of male undergraduate students predicts the proportion of male graduate students (d) which in turn predicts the proportion of male PDs. Each point represents one field. All best‐fit lines represent predicted values from separate LMs. Sample sizes are as follows: mathematics (*n* = 30), physics (*n* = 32), chemistry (*n* = 16), biology (*n* = 328), and psychology (*n* = 27). Because sample sizes differed markedly, error bars represent standard deviations

Our survey received 463 total responses most of which were from female (60% of all PI respondents) biologists (75% of all PIs; Appendix S2, Table S2). The gender‐biased response rate means that results should be interpreted with caution as most fields we surveyed have a majority male researchers (with the exception of psychology) (data from NSERC, 2017). Additionally, response rates were low, <1% of the number of individuals reached based on listserv subscription numbers and the size of departments that were contacted by emails to department chairs. Nonetheless, these responses gave us a reasonably large cross‐sectional data set of male and female PIs with research groups that vary considerably in their reported gender ratios. Fields varied in the number of self‐reported women at each academic stage (all *p* < .001), with the representation of women being generally lowest in physical sciences (Figure 1a, mathematics < physics < chemistry < biology < psychology). The supervisor reported gender of trainees was predicted by the gender of applicants across fields (Figure 1b) for both graduate students (*p* = .001) and PDs (*p* = .01). Analogously, the reported gender composition of undergraduate research students predicted the same metric among graduate students (Figure 1c, *p* = .016) and the reported gender composition of graduate students in turn predicted the gender ratio of PDs (Figure 1d, *p* = .047). Across all fields, the reported representation of men increased at later academic stages (e.g., PD, Figure 1a, *p* = .02), with this effect being particularly pronounced in some fields (math and chemistry, Figure 1a, field * stage interaction: *p* = .003).

### 3.2 Gender patterns within biology

**Figure 2:**
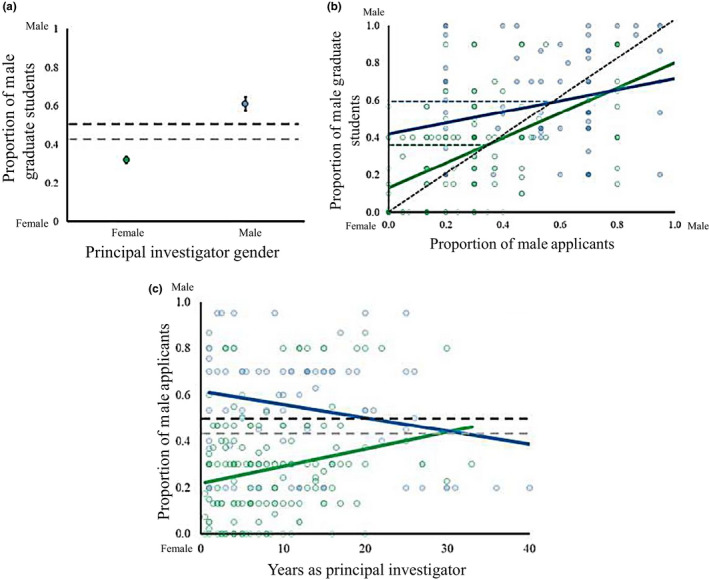
Patterns of trainee and applicant gender in biology. (a) Male PIs (blue) support a greater proportion of male graduate students relative to female PIs (green). Grey and black dashed lines represent the mean proportion of male graduate students and the mean proportion of male graduate students if an equal number of male and female PIs had participated in the survey respectively. Error bars are estimated from a logistic GLM. (b) The proportion of male graduate students in a research group was largely determined by the proportion of male applicants, although this relationship differed for male (blue) and female (green) PIs. The black dashed line shows the 1:1 relationship, indicative of PIs choosing students independent of applicant gender. Point above and below the 1:1 line represent cases where male and female applicants are preferentially selected. Best‐fit lines represent predicted values from a GLM. (c) Male PIs (blue) received more applications from men than female PIs (green). However, this relationship changed depending on the academic experience of the PI, with PI gender having a large effect at an early career stage but being negligible later in their career. In (b) and (c) points were made partly transparent such that more opaque points represent more PIs. Error bars show standard deviations

Within biology, an average research group reported having 43% male graduate students. However, male and female PIs reported supervising 60% and 30% male graduate students, respectively (Figure 2a, *R* ^2^ = .31, *p* < .001). The difference in graduate student gender composition was largely driven by differences in reported applicant gender (Figure 2b, main effect of applicant gender: *p* < .001). However, the relationship between reported gender of applicants and trainees was modified by PI gender, with female PIs more closely tracking applicant gender (slope closer to one in Figure 2b, applicant * PI gender: *p* = .012; *R* ^2^ of complete model is .23). However, our data suggest that female PIs are more likely to select women even when their research groups are not at an equal in gender ratio (when their research group comprised ~35% women versus ~50% for male PIs; dashed lines in Figure 2b which indicate a lower slope in male acceptance to application rates above those values for female and male PIs). The reported gender composition of applicants was in turn driven by PI gender, and an interaction between PI gender and academic experience (Figure 2c; *R* ^2^ of complete model is .32): male PIs reported receiving more graduate school applications from men (main effect of PI gender: *p* < .001), but only when they were hired recently (PI gender * academic experience: *p* = .002).

Gender differences at academic stages other than the graduate stage were less pronounced. The reported gender composition of undergraduate trainees was unaffected by PI gender or experience (all *p* > .25). A given research group had more reported male PDs when more male PDs applied (*p* = .003), but this relationship was unaffected by PI gender (*p* > .3) and neither PI gender nor academic experience predicted the reported gender ratio of PD applicants (both *p* > .4). All results were qualitatively identical after controlling for sample size associated with a given proportion.

### 3.3 Structural equation model within biology

**Figure 3:**
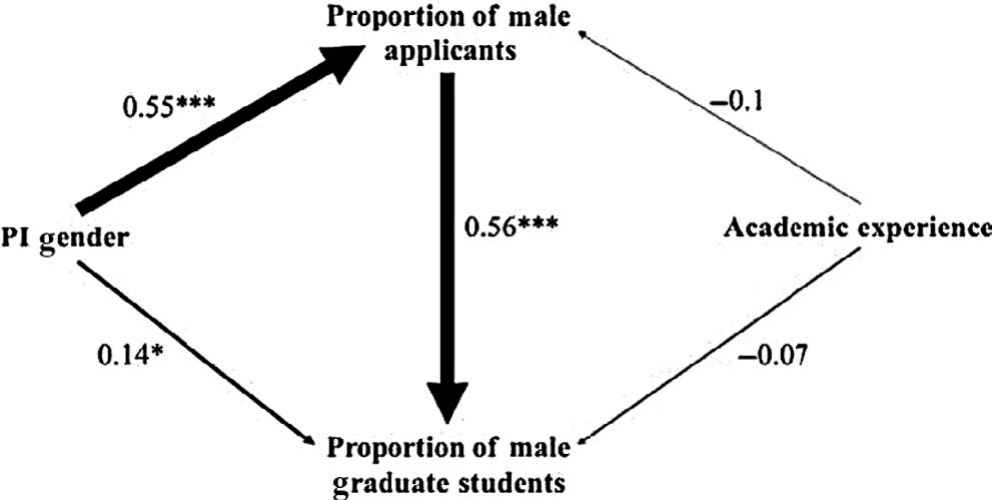
A SEM diagram describing the gender composition of biology graduate students. PI gender predicted the proportion of male graduate students, but largely through its effect on the proportion of male applicants. Numbers represent standardized regression coefficients, and arrows are scaled to the magnitude of the coefficient. Black and red lines represent positive and negative effects, respectively. Significance values: *<.05, **<.01, ***<.001

Our SEM explained the gender composition of students better than models including more/fewer links (ΔAIC = 4.6; *R* ^2^ of final model is .41). PI gender affected the proportion of graduate students who were reportedly male directly (*p* = .03) and indirectly by increasing the number of reported male applicants (both paths *p* < .001), although the latter mechanism was dominant (Figure 3). PI experience reduced the proportion of applicants reported as male; however, this effect was nonsignificant in the absence of interactive terms (both *p* > .4). Overall, our SEM describing the gender composition of graduate students within biology was in agreement with our other analyses.

## 4 DISCUSSION

Our results show that gender differences among research groups exist across and within fields at all stages of academic training. In research groups, women tended to be underrepresented in math, physics, and chemistry, but were equally or overrepresented in biology and psychology (Figure 1a; although absolute gender representation is difficult to interpret from survey data). We attributed measured differences in the representation of women among fields to equivalent differences in the gender composition of applicants (Figure 1). Within biology, male PIs reported having more male‐biased research groups, while the reverse was true for female PIs (Figure 2a); patterns we attributed to both application and acceptance differences (Figure 3). The gender of the reported applicant pool for a given laboratory was in turn predicted by PI gender and years of experience (Figures 2 and 3). We suggest that complex differences in applicant pools and acceptance rates ultimately underlie observed patterns of gender differences among and within fields.

Patterns of gender across fields are consistent with past work showing a lack of women in mathematics and the physical sciences, and suggest that equal gender representation among the professoriate will be unattainable without intervention below the undergraduate level. We found a consistent decline in the number of reported female applicants in math and physical sciences across academic stages (Figure 1a; math < physics < chemistry < biology < psychology), and that this decline was driven by changes in the gender composition of the applicant pool (Figure 1b). Our results corroborate other data showing that physics and biology undergraduates are 19% and >50% female, respectively (Gino, Wilmuth, & Brooks, 2015). However, while the relative representation of women among fields is consistent with past work, we caution against interpreting absolute levels of gender representation (e.g., 50% female) from survey data because the pool of respondents is likely to be biased. Regardless, because so few women are starting undergraduate degrees in the physical sciences, necessarily few women will go on to become professors in those fields (Figure 1c,d; Shaw & Stanton, 2012). This pattern appears to be exacerbated but not driven by declines in the number of women choosing to continue in academia to the level of PD and ultimately professor (Figure 1). Our results then suggest that the gender composition of the applicant pool is a strong factor driving the low representation of women as PIs in the physical sciences (math, physics, and chemistry; Shaw & Stanton, 2012). Conversely, the high representation of women in biology and psychology, and their decline among graduate students and PDs suggests that later career (postgraduate) obstacles are preventing an equal gender representation among professors in these fields (Figure 1a; Miller & Wai, 2015; Sheltzer & Smith, 2014). In sum, among‐field patterns of gender representation in research groups in this study are consistent with past work (e.g., Gino et al., 2015) and appear to be largely driven by differences in the applicant pool, although gender differences in the applicant pool may manifest at different academic stages (e.g., undergraduate to graduate, and graduate to PD; Shaw & Stanton, 2012).

Applicant pools may be male‐ or female‐dominated for several reasons. As discussed earlier, personal and professional considerations can affect men and women differently (Ceci & Williams, 2011; Sheltzer & Smith, 2014). These decisions can be free or constrained, for example by women being more likely to be the dominant caregiver in families with children and more likely to sacrifice their career prospects for those of their partner (relative to men; Ferriman, Lubinski, & Benbow, 2009; Goulden, Mason, & Frasch, 2011Martinez et al., 2007). While these factors undoubtedly play a role, they fail to satisfyingly explain among‐field differences, particularly at the undergraduate level. An intriguing possibility is that women entering undergraduate degrees are less likely to be interested in fields where women hold fewer prominent positions (Drury, Siy, & Cheryan, 2011) and which are perceived as requiring greater natural brilliance (Leslie et al., 2015). The corollary to this idea is that, should enough women become prominent professors in those fields (e.g., math and physics), women entering undergraduate degree may be more likely to apply to those fields, creating the potential for a positive gender feedback‐loop (Leslie et al., 2015). In effect, it is possible that the more women are successful in a field, the more women will want to enter that field.

Within biology, we found that reported application and acceptance rates differed between genders, with PI gender underlying both patterns. Male and female PIs reported having more male and female graduate students, respectively, even after controlling for the skew in the gender of survey participants (Figure 2a). However, the relationship between the reported gender of applicants and the gender of accepted students differed for male and female PIs (Figure 2b). Male PIs had a relatively constant representation of men and women in their laboratories, regardless of the reported proportion of men and women applying. In research groups led by females PIs, the gender ratio within the laboratory tended to closely mirror the gender composition of the reported applicant pool (slope closer to one in Figure 2b). Despite this difference, laboratories headed by male and female PIs appeared to have different gender ratios at which female applicants were disproportionately likely to be accepted (relative to applicant numbers). Female PIs with laboratories with 35% male trainees or more accepted more females than predicted by the applicant pool, while for male PIs this pattern occurred in laboratories that were 50% of trainees were male (dashed lines in Figure 2b). PIs may generate this pattern if they are responding to laboratory gender ratios and adjusting their patterns of acceptance over certain thresholds that differ based on PI gender, or this pattern could be driven by applicants if applicants are more likely to accept offers in laboratories dominated by trainees of the opposite gender. Regardless of the mechanism, both slopes were less than one suggesting that (to differing degrees) research groups led by male and female PIs change which gender disproportionately joins the laboratory, leading to co‐ed research groups within biology.

An important caveat is that we are treating all applicants as equal. It is possible that while male and female PIs treat male and female applicants differently (Figure 2b), that is, this effect is driven not by a simple gender dichotomy, but rather by the interaction of applicant quality and gender (Moss‐Racusin et al., 2012). Whether that decision is conscious or unconscious, whether this change is mediated by differences in the likelihood of applicants accepting positions or PIs changing their selection criteria, and why male and female PIs might approach applicants differently remain open questions.

Research groups led by male and female PIs differed in the gender composition of their students, and at least for early career researchers they also drew from applicant pools that differed in gender composition. Among inexperienced PIs (<10–15 years), men and women reported receiving more applicants from people of the same gender (Figure 2c). Our results contrast those of Sheltzer and Smith (2014) who found that women are underrepresented in the research groups of elite male faculty; however, this may be because they explicitly considered the prestige of the PI rather than their experience. In our study, one possibility is that a lack of information about young PIs may drive a divergence in the gender composition of applicants. Applicants may be more likely to implicitly favor members of their own gender (or generally have implicit bias) if the PI is relatively unknown (e.g., young; Ginther et al., 2011). This hypothesis is supported by the lack of an equivalent pattern among undergraduates or PDs who likely have more information about PIs, either through direct interactions during undergraduate courses or because they are at a more advanced career stage and have a more thorough understanding of their field and interests (Box 1). Explicitly favoring a PI of the same gender may also partly explain applicant gender differences, as several survey participants suggested that women may choose to apply to female PIs under the presumption that female PIs are more likely to be understanding of lifestyle issues. In sum, male and female PIs draw their students from applicant pools that differ in reported gender composition, although this effect disappears later in their careers.

Box 1 **Practical steps to foster gender diversity in research groups**

Unfortunately, there is relatively little research investigating gender dynamics in research groups, making it difficult to provide evidence‐based suggestions. Instead, below we list some *ideas* that *may* address the systemic gender differences found by our study and others. These ideas stem from the comments of reviewers and colleagues. We encourage further study to improve our understanding of academic gender dynamics, with the goal of creating an evidenced‐based framework for addressing such issues.

**Recruitment practices**

Increasing interactions between prospective research students and faculty may be key in increasing the diversity of the applicant pool to particular laboratories, particularly the laboratories of junior faculty.

1. Institutions (universities and departments) can support open houses or recruitment trips for prospective graduate students early in the application process. Early interactions may be most successful as these can occur while prospective students are still identifying potential mentors.
2. Research conferences can facilitate networking opportunities that bring together faculty and prospective graduate students with a particular emphasis on highlighting the research programs of junior faculty where student familiarity may be lowest.
3. Faculty advising undergraduates and/or graduate students can reflect on the diversity of potential supervisors they recommend students investigate for their next position. Encouragement to consider a faculty member of the opposite gender may broaden the pool of mentors students consider.

**Making it work post‐recruitment**

Increasing gender diversity in research laboratories depends not only on diversifying the applicant pool but also on making sure diverse groups work and students are retained. Creating research group culture that supports this diversity is vital to this part of the process and institutions can play a role by providing resources that support this process.

1. Provide funding for conference or research trip travel that supports research group diversity. Having everyone in a research group bunk in a single hotel room during a research trip can work for some groups, but not all. Increasing gender diversity within research groups can result in increased costs for accommodations at conferences or other research trips but university support can ameliorate this.
2. Support communal activities that are welcoming to all. The opportunity to engage in discussions and interactions outside of the more formal work context is one of the great joys of academic training and helps create a social support system for trainees. Opportunities for social interactions can, however, become exclusionary if the context isn't fully considered (e.g., if all social activities occur after 6 p.m. the parents of young children may be systematically excluded). Universities and departments can provide space and resources for inclusive social events. This can be as simple as a regular coffee hour or making sure not all social events happen in the evening.

Our SEM analysis corroborates our earlier results, showing that within biology both reported application and acceptance differences drive the ultimate gender composition of a research group, and that both mechanisms are altered by PI gender (Figure 3). While both mechanisms affected gender in research groups, the effect of reported applicant differences was four times greater than acceptance differences (standardized coefficients in Figure 3). Ultimately, while male and female PIs did tend to select relatively more same‐gender applicants to join their research group, the larger effect can be attributed to differences in reported applicant gender composition. An important caveat is that students declining postacceptance or leaving the laboratory group could equally cause gender‐biased laboratory groups, but this would fail to explain the congruence between application and acceptance rates. Nonetheless, the gender‐based decisions of applicants seem to shape the gender composition of research groups. On a practical level, a conscious consideration of gender biases in networking and when informally recommending supervisors may further reduce gender‐biased application rates.

However, these patterns may have been driven by biased survey responses, since PIs concerned with gender representation may be more likely to respond to surveys that ask questions about laboratory gender composition (relative to PIs who seldom consider gender as an important factor when assembling their research group). Indeed, we observed a female bias in respondents (60% were female), although researchers in all fields we examined except psychology are predominately male (NSERC, 2017). These potential biases would be expected, however, to decrease the effects of PI gender on laboratory composition, suggesting that our results are conservative estimates of these effects. Despite this potential bias, there were still marked differences in gender composition between laboratory groups led by men and women, suggesting that either this pattern is exceedingly strong, or that respondent bias was a relatively minor factor.

An important and nontrivial question is why do gender differences among or within fields matter? Certainly many will agree that equal gender representation is an intrinsic and moral goal. However, are there concrete consequences of differences in gender composition? We expect that there are many, but we highlight those with greatest implications for academia and society. Given that men and women are equally competent, the best possible pool of young scientists would be drawn from fields and research groups that do not discriminate based on gender. This may be particularly important given the projected shortfall in the number of young scientists in the coming decades (Moss‐Racusin et al., 2012). Given the importance of science for the advancement of society, it is important to train enough scientists while drawing from the best possible pool of students. Gender differences in training of qualified students may be detrimental for both academia and society, and we suggest that future work investigates the consequences of these gender differences. In addition to the role of academic laboratories in training future scientists, there is evidence that mixed‐gendered groups produce higher quality science (Campbell, Mehtani, Dozier, & Rinehart, 2013). We may therefore conclude that gender equity in laboratory groups could serve not simply to increase the role of women in science, but would likely be a benefit for all involved, facilitating the production of higher quality work. While there is no prescription that laboratory groups need to have even gender ratios in order to be successful, and given the limited numbers of trainees in most laboratories variation from this at any one time is likely to be the norm, gender diversity appears to yield distinct rewards.

The continued advancement of science, societies, and our knowledge of the world depend on recruiting the best scientists, from a diverse pool of potential scientists without barriers based on gender (or other factors we did not explore here). We have shown that while hiring biases undoubtedly exist, the gender composition of reported applicant pools is the dominant driver of gender differences among trainees (Figures 1, 2, 3) and potentially professors. These patterns are consistent both across the sciences (Figure 1), as well as within a single field (biology; Figures 2 and 3). Crucially, PI gender can itself affect the gender composition of the reported applicant pool (Figure 2c), suggesting that feedbacks between the prominence of women and the gender of applicants may underlie differences in the gender composition of research groups (Leslie et al., 2015). For institutions such as universities, this suggests that programs aimed at fostering gender diversity in their trainees may be most successful at the recruitment stage. One approach might be to encourage interactions between prospective trainees and early career faculty to enhance their familiarity with the research programs of PIs they may not have considered as mentors. This could act to equalize the gender ratio of applicants, particularly for early career PIs (Figure 2c). Simple and pragmatic solutions, such as increased networking and a knowledge of unconscious bias at the personal and institutional level, would likely reduce patterns of gender bias (See Box 1 for specific recommendations). Ultimately, only by addressing issues of gender representation at the applicant level can we hope to eliminate gender differences in the sciences, fostering diverse and productive research groups and allowing for the strongest possible expansion of the scientific workforce.

## CONFLICT OF INTEREST

None declared.

## AUTHOR CONTRIBUTIONS

**Denon Start**: Conceptualization (equal); data curation (equal); formal analysis (equal); investigation (equal); methodology (equal); project administration (equal); visualization (equal); writing – original draft (equal); writing – review & editing (equal). **Shannon McCauley**: Conceptualization (equal); data curation (equal); investigation (equal); methodology (equal); project administration (equal); writing – review & editing (equal).

## Supporting information

Appendix S1

Appendix S2

## Data Availability

Owing to ethical constraints, we are unable to make available the complete data set, although extensive summary statistics are available in supplementary files.
