# Supplementary material for: Gender underlies the formation of STEM research groups
Source: Ecol Evol. 2020 Apr 1;10(9):3834–43. doi: 10.1002/ece3.6188 (PMC7244806; doi:10.1002/ece3.6188)
Supplement: Supplementary file 2 — Appendix S2 [file ECE3-10-3834-s002.docx]

**Appendix S2: Survey Summary**

**Table S2:** Average Proportion of male applicants and trainees by PI gender for all fields.

| PI Gender | Applicants | | Current Trainees | | |
| --- | --- | --- | --- | --- | --- |
|  | Graduate Students | PDs | Undergraduate Students | Graduate Students | PDs |
| Female (n=275) | 0.46 | 0.45 | 0.37 | 0.40 | 0.40 |
| Male (n=175) | 0.39 | 0.57 | 0.39 | 0.48 | 0.57 |
| Non-binary (n=1) | 0.5 | NA | NA | 0.5 | NA |

**Table S3:** The departmental affiliations of PIs which we categorized into distinct fields.

| **Field** | **Departmental Affiliation** |
| --- | --- |
| Asian Studies (n=1) | Asian Studies |
| Biology (n=328) | Agriculture and Natural Resources  Animal Behavior, Ecology, and Conservation  Aquatic and Fisheries Sciences  Bioagricultural Sciences  Molecular Biology  Biological Sciences  Biology  Botany  Center for Genes, Environment, and Health  College of Earth, Ocean, and Environment  Ecology  Ecology and Evolutionary Biology  Ecology and Evolution  Environmental Sciences  Environmental Studies  Forest and Environmental Sciences  Genetics  Genetics and Bioinformatics  Genome Science  Cell Science  Human Ecology  Marine Science  Microbiology  Natural Resource Ecology  Natural Resource Management  Plant Biology  Systems Biology  Wildlife Ecology and Conservation  Wildlife and Fisheries Sciences |
| Geography (n=1) | Geography |
| Chemistry (n=16) | Chemistry |
| Computer Science (n=2) | Computer Science |
| Mathematics (n=30) | Mathematics |
| Physics (n=32) | Physics  Medical Physics  Physics and Astronomy |
| Psychology (n=27) | Psychology  Psychiatry and Psychology  Psychology and Neutroscience |
