# Supplementary material for: Gender underlies the formation of STEM research groups
Source: Ecol Evol. 2020 Apr 1;10(9):3834–43. doi: 10.1002/ece3.6188 (PMC7244806; doi:10.1002/ece3.6188)
Supplement: Supplementary file 1 — Appendix S1 [file ECE3-10-3834-s001.docx]

**Appendix S1: Survey Materials**

*Email Sent to Prospective Respondents:*

Dear Professor,

We are writing to ask for your participation in a short online survey aimed at investigating patterns of gender across academic fields, the link to which can be found below. Please only respond to the survey if you are a principal investigator (with or without graduate students). All personal information including the name of the organization with which you are affiliated will be considered confidential. If you have any questions please contact Denon Start ([denon.start@mail.utoronto.ca](mailto:denon.start@mail.utoronto.ca)) or Dr. Shannon McCauley (Shannon.mccauley@utoronto.ca) for further information. You can find additional information about our study attached.

GO TO SURVEY: <https://www.surveymonkey.com/r/NRQG5VC>

Thank you for your participation.

Denon Start & Dr. Shannon McCauley

*A Table with Survey Questions and Possible Answers:*

**Table S1:** A list of survey question and the associated possible answers. “Other” denotes a blank field where participants can fill in any value or words. The final questions includes a space for other comments.

| **Question** | **Answers** |
| --- | --- |
| To which institution(s) do you belong? | Other |
| To which department(s) do you belong? | Biology, Chemistry, Physics, Mathematics, Other |
| What is your current position? | Professor, Associate Professor, Assistant  Professor, Research Scientist, Other |
| How many years have you been a professor/PI in any capacity? | Other |
| Do you identify as male, female, or non-binary? | Male, Female, Non-binary, Other |
| As what gender did you PhD supervisor identify? | Male, Female, Non-binary, Other |
| How many MALE applicants did you consider for graduate school in the last year? | 0, 1, 2, 3, 4, Other |
| How many FEMALE applicants did you consider for graduate school in the last year? | 0, 1, 2, 3, 4, Other |
| How many MALE students did your research group accept to graduate school in the last year? | 0, 1, 2, 3, 4, Other |
| How many FEMALE students did your research group accept to graduate school in the last year? | 0, 1, 2, 3, 4, Other |
| How many applications for post-doctoral work did you consider from MALES in the last year? | 0, 1, 2, 3, 4, Other |
| How many applications for post-doctoral work did you consider from FEMALES in the last year? | 0, 1, 2, 3, 4, Other |
| How many MALE post-doctoral fellows did you accept in the last year? | 0, 1, 2, 3, 4, Other |
| How many FEMALE post-doctoral fellows did you accept in the last year? | 0, 1, 2, 3, 4, Other |
